# SCID小鼠宣威肺癌原位模型的构建及生物学特性研究

**DOI:** 10.3779/j.issn.1009-3419.2012.08.01

**Published:** 2012-08-20

**Authors:** 永春 周, 艳 陈, 熙才 王, 馨 刘, 湖涛 施, 乾 姚, 从国 金, 治平 伍, 云超 黄

**Affiliations:** 1 650118 昆明，昆明医科大学第三附属医院，云南省肿瘤研究所 Yunnan Tumor Research Institute, the Third Affiliated Hospital of Kunming Medical University, Kunming 650118, China; 2 650228 昆明，昆明同仁医院放射科 Department of Radiology, Kunming Tongren Hospital, Kunming 650228, China

**Keywords:** 宣威, 肺肿瘤, 原位模型, SCID小鼠, Xuanwei, Lung neoplasms, Orthotopic model, SCID mouse

## Abstract

**背景与目的:**

宣威女性肺癌发病率居全国首位，亟待深入探讨其发病机制。本研究拟建立SCID小鼠原位宣威肺癌模型，为该病的深入研究提供实验平台。

**方法:**

将宣威肺癌细胞XWLC-05分别以高低剂量接种于SCID小鼠肺原位，并与皮下移植瘤比较，观察成瘤率、成瘤特性、自发性转移及生存情况。

**结果:**

原位移植高低剂量组的成瘤率分别为81%和83%，其中高剂量组接种后13天小鼠出现恶液质、对侧肺及胸腔的广泛粘连，无远处转移；低剂量组接种后25天小鼠出现恶液质及远处转移。皮下移植高低剂量组成瘤率分别为100%及94.5%，无远处转移。原位移植组组内及皮下与原位移植组组间的转移率存在统计学差异（*P* < 0.05）。两组组内和组间的生存率比较有统计学差异（*P* < 0.001）。

**结论:**

成功建立了宣威肺癌的SCID小鼠原位动物模型，为该病的深入研究奠定了实验基础。

## Abstract (en)

**Background and objective:**

The incidence of Xuanwei lung cancer ranks first in China, and its pathogenesis requires in-depth investigation. This study aims to establish an orthotopic Xuanwei lung cancer severe combined immunodeficiency (SCID) mouse model and to provide a basic experimental platform for further study.

**Methods:**

The Xuanwei lung cancer cell line XWLC-05 was inoculated into the lung tissue of SCID mice in high and low doses. The tumor formation rates, tumor characteristics, spontaneous metastases, and survival times of the mice were observed, taking a subcutaneously transplanted tumor as control.

**Results:**

The tumor formation rates of the orthotopic transplantation of lung cancer cells in high and low doses were 81% and 83%, respectively, among which mice in the high-dose group appeared cachectic on day 13. Extensive invasion and adhesion were observed in the contralateral lung and thoracic cavity, but no distant metastasis was exhibited. Mice with low-dose cells in the orthotopic transplantation group appeared cachectic and distant metastasis occurred on day 25. The tumor formation rates in the subcutaneous inoculation group by the high and low doses of cells were 100% and 94.5%, respectively, and no distant metastasis was observed. The rate of metastasis within the orthotopic transplantation group and between the orthotopic and subcutaneous inoculation groups showed a significant difference (*P* < 0.05). A significant difference was indicated by the survival rate within and between the groups (*P* < 0.001).

**Conclusion:**

We successfully established an orthotopic XWLC SCID mouse model, which lays the foundation for a more in-depth study.

宣威地区位于中国滇东北珠江流域源头，当地女性肺癌的调整死亡率是全国平均水平的8倍，已成为严重危害人民群众生命健康的疾病^[1]^，而导致肺癌患者治疗失败和死亡的主要原因是肿瘤侵袭和转移^[2]^。因此，建立可靠的原位动物模型对深入研究宣威肺癌的发生和转移机制具有重要的作用。与肿瘤的异位移植相比，原位模型的建立更能模拟肺癌在人体内的生物学特性，为研究提供相对客观的依据^[3-5]^。本研究将XWLC-05宣威肺癌细胞移植到联合免疫缺陷鼠（severe combined immunodeficiency mice, SCID）肺组织，建立肺癌原位移植模型，以探讨其成瘤及转移的生物学特性。

## 1 材料与方法

### 1.1 实验动物

4周-5周龄雌雄随机SCID小鼠72只（购自北京维通利华公司公司），许可证号为SCXK（京）2012-0001，合格证号2012-0001，体重为16 g-19.5 g，置于层流式超净架内的有机玻璃饲养盒内（6只/盒）。饲养环境为无特定病原体级（specefic pathogen free, SPF），无菌标准饲料及饮水，环境温度25 ℃-27 ℃，相对湿度为45%-50%，12 h光暗周期。动物无菌培养房由昆明医科大学重点实验室提供。接种前小鼠在动物房培养观察1周以保证接种前生长状况正常。定期给小鼠添加饲料、灭菌水及更换垫料，每两天对小鼠进行体重称量，观察其生长及精神状况。

### 1.2 细胞株

宣威肺癌细胞株XWLC-05由昆明医科大学第一附属医院提供，在本实验室常规传代培养，实验用细胞为第16代。

### 1.3 细胞生长曲线绘制及细胞悬液的制备

XWLC-05细胞培养于含10%胎牛血清的RPMI1640培养液中，于37 ℃、含5%CO_2_的培养箱内培养。在满足动物接种所需细胞量的同时，控制传代次数，以保证细胞活力。取对数生长期的各组细胞以1.0×10^2^个/孔接种于96孔培养板上，每组细胞设5个重复孔，放置于37 ℃、含5%CO_2_浓度的细胞培养箱中孵育4 h、2 d、5 d、7 d。按细胞增殖检测试剂盒操作说明书进行MTS（3-(4, 5-dimethylthiazol-2-yl)-5-(3-carboxymethoxyphenyl)-2-(4-sulfophenyl)-2H-tetrazolium, inner salt）比色法检测，培养结束后，每孔加入20 μL MTS/PMS混合液，反应1 h后，用酶标仪（Bio-Rad公司）测定490 nm处吸光度值（optical delnsity, OD），实验重复3次。根据计数结果以OD值为纵坐标，以时间为横坐标绘制生长曲线^[6]^，取第4天处于对数生长期的XWLC-05细胞（图 1），以0.25%的胰酶消化，收集细胞，离心去上清，用PBS洗涤两次，台盼蓝染色确定细胞活力大于90%，将细胞悬浮于生理盐水中，调整细胞浓度为2×10^6^/mL（用于低剂量组接种）及1×10^7^/mL（用于高剂量组接种）。

**1:**
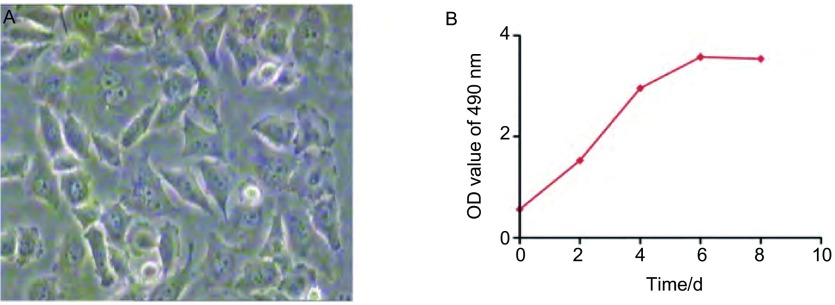
XWLC-05细胞的生长状态。A：光镜下处于对数生长期的XWLC-05细胞（× 200）；B：XWLC-05细胞生长曲线。 Growth state of XWLC-05 cells. A: Cellular morphology of XWLC-05 cells (×200); B: Growth curve of XWLC-05 cells.

### 1.4 动物模型的建立

#### 1.4.1 实验动物分组

将72只小鼠随机分为4组，每组18只。皮下移植：取其中两组分别进行高剂量及低剂量接种，每组中的8只用于观察生存，10只用于监测肿瘤及转移情况。原位移植：取其中两组分别进行高剂量及低剂量接种，每组中的8只用于观察生存，10只用于监测肿瘤及转移情况。

#### 1.4.2 原位移植

用依托米酯注射液，按体重0.01 mL/g腹腔内注射，小鼠经兴奋烦躁期后进入麻醉状态，仰卧固定，前胸壁酒精消毒，在左腋前线肋弓上约1.5 cm处作一约5 mm的小切口，分离皮肤及皮下组织暴露至胸壁，分别取高剂量及低剂量组50 μL（即5×10^5^/只和1×10^5^/只）细胞悬液与50 μL Matrigel混匀，在快凝固之前以胰岛素注射针将混合悬液注入小鼠左肺，进针深度约3 mm，注射完后停针5 s，拔针后用眼科铲针缝合切口，再次酒精消毒切口。

#### 1.4.3 皮下移植

酒精消毒小鼠右腋下皮肤，针尖挑起皮肤，将针插入皮下注射50 μL细胞悬液，稍作停留后拔针，用棉签轻轻压住腋下针孔，以防止细胞悬液漏出。

### 1.5 移植瘤生长的观察及测量

#### 1.5.1 一般观察及测量

定期观察SCID小鼠的精神、饮食、排便、体重和活动等情况，当出现明显体重下降、食欲不振、饮水量减少、精神萎靡、皮肤干燥及呼吸困难等现象时，脱颈处死并解剖小鼠，观察肿瘤形成及转移情况。原位移植组：取出原位移植瘤及肿大淋巴结、肝、脾、胰、肺、肾及脑组织，用10%甲醛固定、石蜡切片、HE染色后行光镜下病理组织学检查。小鼠处死前用亚甲蓝0.5 mL做足垫及掌垫注射，以示踪淋巴结。皮下移植组：于肿瘤形成后第3天开始测量肿瘤大小，用游标卡尺测量肿瘤长径及短径，以公式V=a^2^bπ/2（a为短径，b为长径）计算肿瘤大小，同时取出肿大淋巴结、肝、脾、胰、肺、肾及脑组织做病理组织学检查。

#### 1.5.2 CT扫描

小鼠处死前运用东芝64排CT对小鼠胸部进行螺旋扫描，以层厚2 mm、层间距2 mm分别重建出横断位及冠状位图像。

### 1.6 统计学处理

采用SPSS 17.0软件进行统计分析，率的比较采用卡方检验或*Fisher*精确检验，采用*Kaplan-Meier*法进行生存分析。以*P* < 0.05为差异有统计学意义。

## 2 结果

### 2.1 一般观察

小鼠于麻醉后30 min左右开始苏醒，1 h后能够恢复活动。实验过程中原位移植高剂量组小鼠死亡2只，因手术操作不当导致气胸死亡。原位移植高低剂量组小鼠分别于第13天和第25天出现消瘦、活动减少、饮食减少及倦呆乏力等恶液质表现；皮下高剂量接种组在第29天出现以上症状；皮下低剂量接种组状态良好，恶液质出现较晚，最长生存期可达53天。

### 2.2 肿瘤形成情况

#### 2.2.1 原位移植组

高低剂量组的成瘤率分别为81%和83%，差异无统计学意义（*P*=0.932），其中高剂量组于接种后第13天解剖就有肿瘤形成，且出现对侧肺、胸膜腔及腋下淋巴结的广泛转移（图 2A，图 2B）；低剂量组于接种后第25天解剖有肺部原位肿瘤形成，肿瘤质地中等，白色，浸润周围组织，切面呈鱼肉状，符合肺癌的病理组织特点（图 2C，图 2D）。低剂量组淋巴结、对侧肺、肝、脑、骨及肾的转移率分别为90%、80%、60%、50%、70%、60%，与原位高剂量组相比，除淋巴结（*P*=0.50）和对侧肺（*P*=0.709）外，肝（*P* < 0.01）、脑（*P* < 0.01）、骨（*P* < 0.01）和肾（*P* < 0.01）两组的转移率差异有统计学意义（表 1）。各部位转移病灶均经病理组织学检查证实（图 3）。CT扫描提示原位低剂量接种组除有肺原位成瘤外，其中3只小鼠还有胸腔积液的形成（图 4）。

**2:**
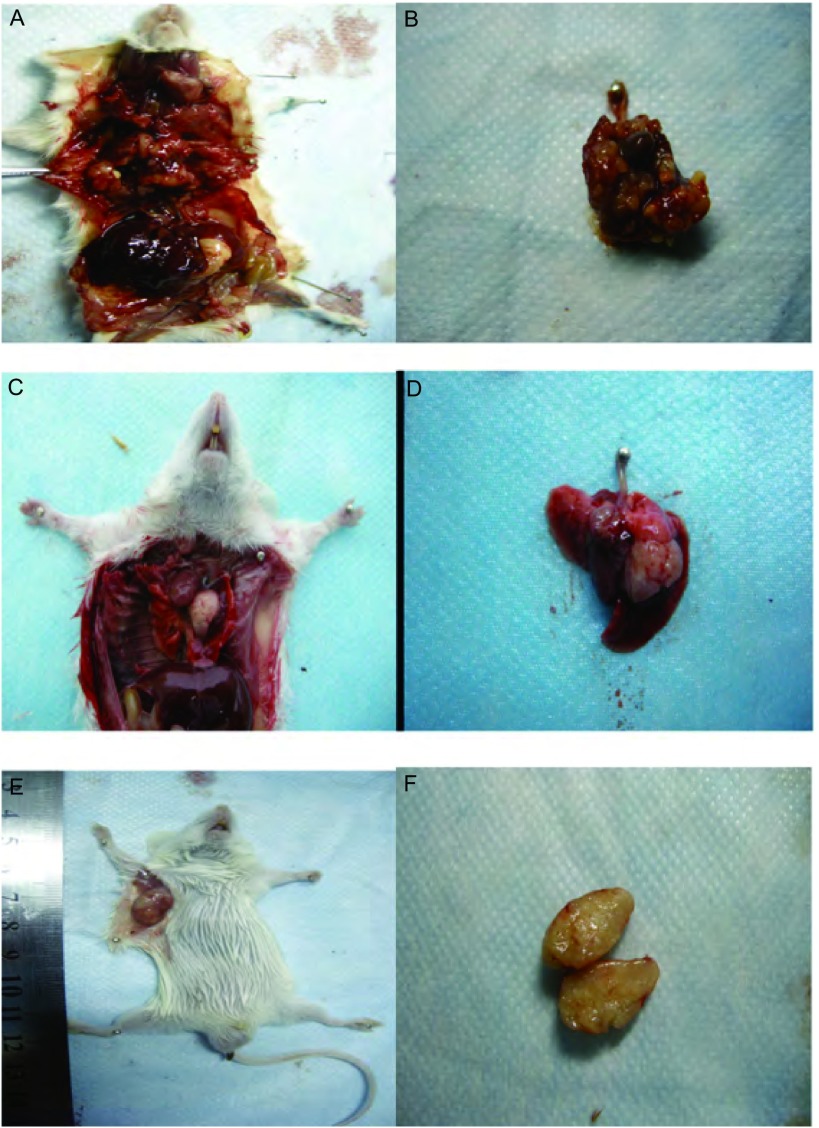
原位及皮下移植瘤模型。A，B：原位高剂量XWLC-05细胞接种组小鼠显示双侧肺和胸膜腔的广泛浸润转移；C，D：原位低剂量XWLC-05细胞接种组小鼠显示左肺境界清楚的肿瘤形成；E，F：皮下低剂量XWLC-05细胞接种组小鼠显示皮下肿瘤的形成及肿瘤切面。 Orthotopic transplantation and subcutaneous xenograft model. A, B: Mouse of orthotopic transplantation with high dose of XWLC-05 cells in left lung shows serious infiltration in bilateral lungs and extensive pleural metastases; C, D: Mouse of orthotopic transplantation with low dose of XWLC-05 cells in left lung shows a clear boundary tumor in left lung; E, F: Mouse of subcutaneous inoculation with low dose of XWLC-05 cells: tumor *in situ* and its section view.

**1:** 不同细胞剂量各移植组间转移率的比较 Comparison of metastasis rate among different groups

Metastases sites	Orthotopic transplantation group [*n*(%)]			Subcutaneous inoculation group [*n*(%)]	
	High dose (*n*=8)	Low dose (*n*=10)		High dose (*n*=10)	Low dose (*n*=9)
Lymph node	7 (87.5)	9 (90)		0	0
Contralateral lung	6 (75)	8 (80)		0	0
Liver	0	6 (60)		0	0
Brain	0	5 (50)		0	0
Bone	0	7 (70)		0	0
Kidney	0	6 (60)		0	0

**3:**
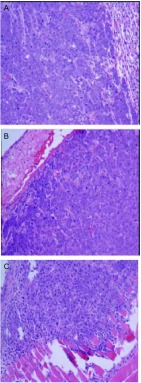
原位低剂量XWLC-05细胞移植组小鼠组织（HE，× 200）。A：左肺肿瘤；B：左锁骨上淋巴结转移性肺癌；C：右胸壁肺癌转移灶。 Representive tissue HE staining picture of mouse received orthotopic transplantation with low dose of XWLC-05 cells (HE, ×200). A: Tumor in left lung; B: Metastasis lymph node above left clavicle; C: The right chest wall metastases.

**4:**
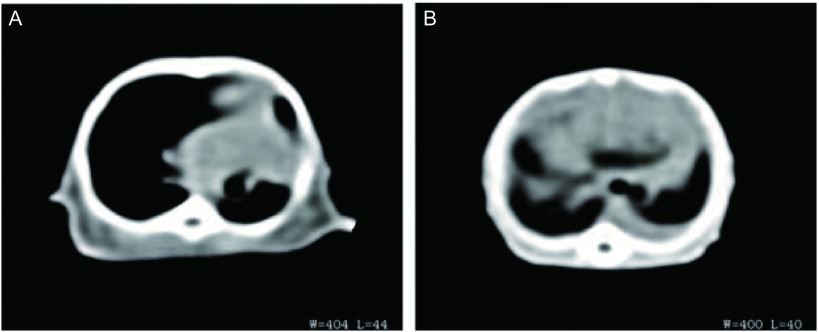
原位低剂量XWLC-05细胞移植小鼠的胸部CT扫描影像。A：左肺下叶形的肿瘤；B：双侧肺门肿块合并左侧胸腔积液。 Representive CT scan images of mouse received orthotopic transplantation with low dose of XWLC-05 cells. A: Tumor formed in left lung lower lobe; B: Bilateral hilar mass with left pleural effusion.

#### 2.2.2 皮下移植组

高剂量组于第7天成瘤，低剂量组于第11天成瘤（其中有1只小鼠因操作不当未形成肿瘤），成瘤率分别为100%及94.5%，差异无统计学意义（*P*=0.791）（图 5），皮下移植高低剂量组与原位移植高低剂量组间的成瘤率比较，差异无统计学意义（*P*=0.365, *P*=0.584）。皮下肿瘤解剖后观察：肿瘤质地中等，白色，部分有薄包膜形成，切面呈鱼肉状，部分肿瘤过大可见中央有红黑色坏死灶（图 2E，图 2F）。高低剂量组均未发现远处转移，与原位高低剂量移植组相比，各部位的转移率差异有统计学意义（*P* < 0.01）（表 1）。

**5:**
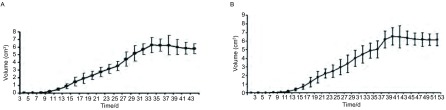
皮下移植瘤生长曲线。A：高细胞剂量组；B：低细胞剂量组。 Growth curve of subcutaneously transplanted tumor. A: High-dose group; B: Low-dose group.

### 2.3 小鼠生存情况

原位移植组高低剂量的中位生存期（17天、27天）明显少于皮下移植组（33天、49天），生存分析显示皮下移植组、原位移植组组间及组内各剂量间的生存率比较差异有统计学意义（*P* < 0.001，图 6）。

**6:**
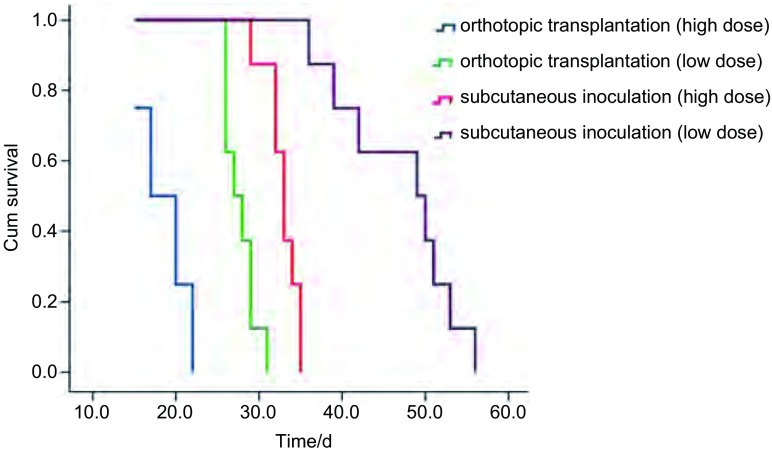
不同移植组各剂量间小鼠生存曲线 Survival curve of mice in different groups

## 3 讨论

肿瘤实验动物模型是人类肿瘤的复制，在肿瘤发病机制研究及治疗药物筛选等领域发挥着重要的作用。目前国内外肿瘤的实验动物模型主要包括以下类型：①自发瘤模型，实验动物未经任何有意识的人工处置，在自然情况下所发生的肿瘤；②诱导模型，用致癌物在动物一定部位或一定器官诱发出的肿瘤。因存在成瘤周期长、饲养动物量大、成瘤率低及耗资大等缺点，以上两类模型多应用于职业暴露或环境污染等相关的肿瘤研究^[7, 8]^，在常规肿瘤研究中很少运用。此外还有转基因动物模型及目前应用最多的移植模型，用肿瘤组织或细胞移植到动物体内所形成的肿瘤动物模型，因其制作简单、成瘤率稳定及成瘤时间短而被各实验室广泛运用^[9]^。在移植瘤模型中既往常用异位皮下移植，该方法虽然成瘤率高，但因受血供及细胞因子等微环境的限制，不能反应肿瘤在人体中的特征^[10]^。因此，近年来多采用原位肿瘤动物模型开展研究，它是指将人类肿瘤细胞或组织移植在动物的相应部位，因其与在人体内具有相同或相近的微环境，从而可获得生物学行为更接近人体原发肿瘤特性的模型。在肺癌的研究中原位动物模型的接种方式又包括了支气管内注射和肺内注射。支气管注射操作困难、成瘤率低及瘤体的大小、数目均不稳定，其运用受到一定的限制^[11]^。而与之相比，肺内注射兼具了操作简单、成瘤率高、成瘤时间短及肿瘤生长环境更符合人体情况等优点。在实验动物的选择上既往多采用的是BALB/c裸鼠，但因其为单免疫系统缺陷，仍有许多人类肿瘤无法在裸鼠体内移植成活^[12]^。本实验中采用的SCID小鼠是严重联合免疫缺陷小鼠，上世纪80年代由美国Bosma等^[13]^改造培殖而成，由于该小鼠为T、B细胞联合缺陷，对移植物免疫排斥较低，因此比单一缺损T淋巴细胞的裸鼠更适于作异体移植。Gemma等^[14]^及Shindo-Okada等^[15]^曾利用PC9、PC14及A549肺腺癌细胞系进行小鼠原位移植，除形成纵隔淋巴结转移外，均未形成远处器官转移。本研究选择云南宣威肺癌细胞株系XWLC-05进行小鼠移植，该细胞系细胞系2007年由昆明医学院病理教研室建立，是目前唯一一株以宣威女性肺癌为来源建立的细胞系，材料取自宣威当地一位68岁的女性肺腺癌患者，病理诊断为右肺中分化腺癌。经实验证明，该细胞系维持了原肿瘤的表型特征，能形成和宣威女性肺癌具有相同形态学特征的裸鼠移植瘤^[16]^，选择该细胞作为体外研究的对象，能最大程度地反映宣威女性肺腺癌的生物学特征。

本次研究将XWLC-05细胞分两组并以不同剂量分别接种于SCID小鼠皮下及肺原位，以观察其成瘤及转移情况。实验结果显示：皮下移植组无论高低剂量，小鼠在生存期内都未形成远处转移灶，考虑由于其周围有结缔组织包裹及生长的微环境缺乏多种与转移相关的细胞因子，故仅能形成局部肿瘤，限制了肿瘤的浸润及转移。因此皮下移植形成的肿瘤不能满足对肺癌转移机制的研究。与皮下移植瘤相比，原位移植具有血液供应丰富及局部微环境更接近人体等优越性，不仅移植成功率高，而且更利于形成转移^[17]^。在本实验的36只原位移植的小鼠中，移植成瘤率均在80%以上，使用5×10^5^/只高剂量细胞组的小鼠于接种后短期既形成肿瘤，且发生胸腔及对侧肺的广泛转移，在尚未形成远处转移病灶前即很快死于呼吸功能衰竭，中位生存期仅有17天，不仅其发病和转移的特点与临床有一定的差异，且小鼠过短的生存期不利于开展转移机制的深入研究；与之相比，使用1×10^5^/只低剂量组细胞原位接种的小鼠生存期相对较长，亦成功形成了原位肿瘤，移植瘤发生局部浸润、淋巴结转移及远处脏器转移，且转移率较高，部分有胸水形成，较好地保持了原始肿瘤的组织结构、细胞形态、功能及生化特征，其转移的发生发展符合临床肺癌患者的特点和规律，因此笔者认为该数量级细胞建立的肿瘤模型对转移机制的研究更为理想。在原位移植中，为避免肿瘤细胞的扩散而不能形成单一的肿瘤，我们将肿瘤细胞与具有粘附性的Matrigel先混合后接种，充分保证了在局部的接种数量。

本研究以宣威肺癌XWLC-05细胞直接接种于SCID小鼠肺内，除对原位构建方法进行了优化外，还从形态学观察、影像学检查、病理学检查及生存统计等层面对移植效果进行了综合评估，成功构建了敏感、稳定及转移特性与人体相符的原位肿瘤动物模型，为宣威肺癌及其他非小细胞肺癌转移机制的研究及抗肿瘤药物的开发奠定了实验基础。
